# Exploring the Role of Surfactants in Enhancing Drug Release from Amorphous Solid Dispersions at Higher Drug Loadings

**DOI:** 10.3390/pharmaceutics13050735

**Published:** 2021-05-17

**Authors:** Sugandha Saboo, Pradnya Bapat, Dana E. Moseson, Umesh S. Kestur, Lynne S. Taylor

**Affiliations:** 1 Department of Industrial and Physical Pharmacy, Purdue University, 575 Stadium Mall Drive, West Lafayette, IN 47907, USA; saboo7@purdue.edu (S.S.); pbapat@purdue.edu (P.B.); dmoseson@purdue.edu (D.E.M.); 2 Oral Formulation Sciences and Technology, Merck & Co., Inc., Rahway, NJ 07065, USA; 3 Drug Product Development, Bristol-Myers Squibb Company, One Squib Drive, New Brunswick, NJ 08903, USA; umesh.kestur@bms.com

**Keywords:** amorphous solid dispersions, drug release, polymer release, congruent, surfactant, drug loading, phase behavior

## Abstract

To reduce the dosage size of amorphous solid dispersion (ASD)-based formulations, it is of interest to devise formulation strategies that allow increased drug loading (DL) without compromising dissolution performance. The aim of this study was to explore how surfactant addition impacts drug release as a function of drug loading from a ternary ASD, using felodipine as a model poorly soluble compound. The addition of 5% TPGS (d-α-tocopheryl polyethylene glycol 1000 succinate, a surfactant) to felodipine-polyvinylpyrrolidone/vinyl acetate ASDs was found to facilitate rapid and congruent (i.e., simultaneous) release of drug and polymer at higher DLs relative to binary ASDs (drug and polymer only). For binary ASDs, good release was observed for DLs up to <20% DL; this increased to 35% DL with surfactant. Microstructure evolution in ASD films following exposure to 100% relative humidity was studied using atomic force microscopy coupled with nanoscale infrared imaging. The formation of discrete, spherical drug-rich domains in the presence of surfactant appeared to be linked to systems showing congruent and rapid release of drug and polymer. In contrast, a contiguous drug-rich phase was formed for systems without surfactant at higher DLs. This study supports the addition of surfactant to ASD formulations as a strategy to increase DL without compromising release. Furthermore, insights into the potential role of surfactant in altering ASD release mechanisms are provided.

## 1. Introduction

An increasing number of drug candidates emerging from drug discovery pipelines are poorly water soluble [1,2]. Amorphous drugs offer increased transient solubility compared to their crystalline counterparts; however, this solubility advantage is often not sufficient to deliver high dose drugs [3,4]. Therefore, the dissolution advantage derived from polymer-controlled dissolution of amorphous solid dispersions (ASDs), where drug is molecularly dispersed in a hydrophilic polymer matrix, is gaining increased attention from drug product formulators and researchers [5,6]. The dissolution rate of a hydrophilic polymer is many times higher compared to that of a lipophilic drug. Consequently, drug release from an ASD, when polymer-controlled, is typically relatively rapid and results in a highly supersaturated solution with a drug concentration that exceeds the amorphous solubility [7,8]. The drug in excess of amorphous solubility phase separates to form in situ amorphous drug-rich nanoparticles that are dispersed in the bulk aqueous phase, which is a phenomenon referred to as liquid–liquid phase separation [9]. These nanoparticles, owing to their small size (<1 µm), provide additional dissolution benefits and, in turn, a bioavailability advantage [10,11,12]. However, the exact mechanism of the formation of these nanoparticles as well as their role in bioavailability enhancement is still under debate [10,11,13,14,15].

The polymer-controlled dissolution advantage of ASDs as well as the resulting amorphous nanoparticles formation is typically only realized up to the limit of congruency (LoC, the highest drug loading up to which the drug and polymer release congruently, i.e., at the same rate), which often corresponds to a low drug loading limit, in particular in the case of polyvinylpyrrolidone/vinyl acetate-based ASDs [7,8,16,17]. In such cases, either the drug loading must be kept low, or formulation strategies need to be devised to improve the LoC. Maintaining a low drug loading has an inherent disadvantage for high-dose drugs, resulting in large or multiple dosage units, decreasing patient compliance. Recently, it was noted that the choice of polymer might impact the LoC, with an accompanying “trade-off”, whereby ASDs with relatively hydrophobic polymers showed higher LoCs at the expense of slower release rates relative to ASDs with more hydrophilic polymers [4]. Therefore, achieving fast and complete release from ASDs incorporating hydrophilic polymers at reasonably high drug loads remains a desirable outcome.

Ternary ASDs incorporating surfactants show altered performance relative to their binary counterparts. Improved wettability [18,19,20], crystallization inhibition (both in solid and solution state) [21,22], dissolution enhancement (with and without solubilization) [19,23], and enhanced formation as well as stabilization of amorphous drug-rich nanoparticles [16,23,24], are some of the advantages that have been observed with ternary ASDs containing surfactants. However, in some cases, adding surfactants to the ASDs has been demonstrated to elevate crystallization risk, both from solid and solution states [20,21,25]. For instance, Baghel et al. reported that the addition of sodium dodecyl sulfate and poloxamer 188 reduced the physical stability and dissolution of polyvinylpyrrolidone and hydroxypropyl methylcellulose-based ASDs by promoting crystallization [25]. In two separate studies with itraconazole ternary ASDs, the inclusion of d-α-tocopheryl polyethylene glycol 1000 succinate (TPGS) showed a different impact whereby TPGS showed synergistic precipitation inhibition as part of an HPMCAS-based ternary ASD, while the addition of TPGS resulted in phase separation and crystallization when included as part of a PVPVA-based ternary ASD [26,27]. Nevertheless, provided that crystallization can be prevented by judicious choice of the polymer–surfactant combination for a given drug, or for instances where the drug is a slow crystallizer, ternary ASDs with surfactants can provide release advantages. In a recent publication, Que et al. found that the LoC of ledipasvir–PVPVA ASDs was increased by some but not all of the surfactants evaluated, although the mechanism behind the enhanced release remains elusive [16].

**Figure 1:**
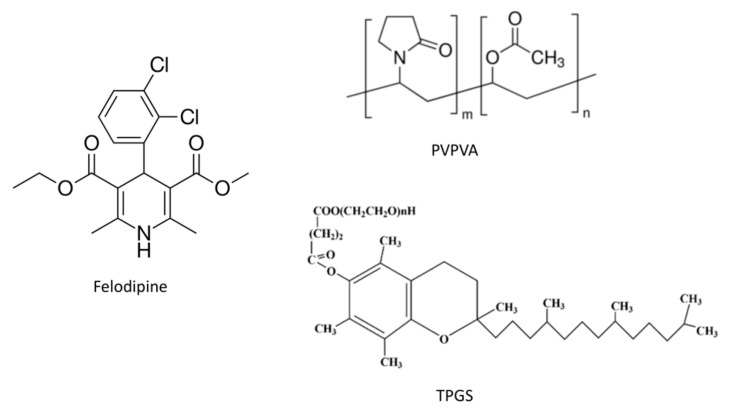
Chemical structures of felodipine (drug), PVPVA (polymer), and TPGS (surfactant).

The goals of the current study were two-fold: first, to demonstrate that the LoC of a model ASD system could be increased by the inclusion of a surfactant, evaluating the impact of surfactant level and second, to probe the mechanism by which the surfactant increases the LoC. Felodipine, a biopharmaceutical classification system class II drug, which has been extensively studied in ASD formulations, was chosen as the model drug [28,29,30]. Polyvinylpyrrolidone/vinyl acetate (PVPVA), a hydrophilic polymer, which has been widely utilized in marketed ASD-based pharmaceutical products, was selected as the model polymer [6]. TPGS, a pharmaceutically relevant surfactant present in commercial ASD formulations, Viekira Pak and Belsomra, was used to formulate ternary systems. Figure 1 shows the structures of drug, polymer, and surfactant used in this study. Table S1 shows the physicochemical and thermodynamic properties of felodipine. In a previous study, we found that the LoC of felodipine-PVPVA ASDs is only 15% DL [4]. Herein, we prepared felodipine-PVPVA-TPGS ASDs, and their release performance was examined using surface-normalized dissolution at different drug loadings and various TPGS levels. The impact of surfactant incorporation on amorphous drug-rich nanoparticles generation was also investigated. The glass transition temperatures of ASDs were measured using modulated differential scanning calorimetry (DSC). Partially dissolved ASD tablet surfaces were examined using microcomputed tomography (microCT). Atomic force microscopy coupled with nanoscale infrared spectroscopy (AFM-nanoIR) was employed to investigate the evolution of microstructure and to characterize the local compositions within ASD films following phase separation induced by storage at high relative humidity.

## 2. Materials and Methods

### 2.1. Materials

Felodipine (Fel) was purchased from Chemshuttle (Jiangsu, China). Polyvinylpyrrolidone/vinyl acetate (PVPVA, Kollidon VA 64) was obtained from the BASF Corporation (Ludwigshafen, Germany). d-α-tocopheryl polyethylene glycol 1000 succinate (TPGS) was purchased from Sigma-Aldrich (St. Louis, MO, USA). Methanol and dichloromethane were purchased from Mallinckrodt Baker (Phillipsburg, NJ, USA). The aqueous media used in all experiments was kept constant, i.e., 100 mM pH 6.8 phosphate buffer, which was prepared by dissolving 7.06 g of monosodium phosphate monohydrate and 6.94 g of sodium phosphate dibasic anhydrous in 1 L of deionized water.

### 2.2. Methods

#### 2.2.1. Critical Micelle Concentration Measurement

The critical micelle concentration (CMC) of d-α-tocopheryl polyethylene glycol 1000 succinate (TPGS) was determined by two different methods: first, based on felodipine solubility measurements and second, using fluorescence measurements after spiking a fluorescence probe, pyrene, into the medium of interest. In the former method, an excess amount of crystalline felodipine was equilibrated in the dissolution buffer with different amounts of TPGS for 48 h at 37 °C. Then, samples were centrifuged at 151,000× *g* for 30 min (37 °C) using a swinging bucket rotor (SW 41Ti) in an Optima L-100XP ultracentrifuge (Beckman Coulter Inc., Brea, CA, USA). The supernatant was analyzed by high-performance liquid chromatography method as described previously [4]. Briefly, an Ascentis Express C18 column (Sigma-Aldrich, St. Louis, MO, USA) with a mobile phase of 70% acetonitrile and 30% water (*v/v*) at a flow rate of 0.5 mL/min was used. Appropriate sample dilutions were carried out to match the organic to aqueous ratio of the analyte with the mobile phase composition. The TPGS concentration where an abrupt increase in felodipine solubility occurred was determined as the CMC. In the fluorescence-based method, the fluorescence probe, pyrene, was used as a surrogate for the hydrophobic drug and spiked into the dissolution buffer (0.01% *w/v*) containing varied TPGS concentrations to measure the CMC. Samples were analyzed using a Shimadzu spectrofluorophotometer RF-5301PC (Shimadzu Scientific Instruments Inc., Columbia, MD, USA) at an excitation wavelength of 332 nm. The samples were pre-equilibrated at 37 °C before measurements. The ratio between fluorescence emission intensity at 372 nm (I_1_) and 383 nm (I_3_) was plotted against the concentration of TPGS. An abrupt change in the ratio of I_1_/I_3_ signified a change in polarity of the probe environment, and the corresponding TPGS concentration was determined as its CMC. All CMC determination experiments were repeated for the dissolution buffer containing 900 μg/mL pre-dissolved PVPVA.

#### 2.2.2. Amorphous Solubility Measurement

The crystalline and amorphous solubilities of felodipine in 100 mM pH 6.8 phosphate buffer have been determined previously, and they are 1.3 and 8 µg/mL, respectively [4]. In this study, the amorphous solubility measurement of felodipine was repeated in the same medium in the presence of 900 μg/mL PVPVA to understand the impact of high polymer concentration, if any, on the maximum achievable free drug concentration in solution in the absence of crystallization. The previously published method was utilized for amorphous solubility determination [4].

#### 2.2.3. Preparation of Ternary Amorphous Solid Dispersions (ASDs)

The composition of binary amorphous solid dispersions is typically expressed in weight percent of drug and polymer. In order to understand the impact of a third component, i.e., a surfactant, on the ASD dissolution performance as a function of drug loading, we chose to vary the TPGS content (2%, 5%, and 10%) on the weight percent basis out of the polymer composition and prepared ternary amorphous solid dispersions of felodipine, PVPVA, and TPGS at different drug loadings, as shown in Table S2. Percent (%) refers to weight percent in this work unless otherwise specified. ASDs were prepared by rotary evaporation using a Buchi Rotavapor-R (New Castle, DE, USA) equipped with a Yamato BM-200 water bath which was set at 45 °C. Stock solutions were prepared for rotary evaporation by dissolving components at the desired ratio in a 1:1 *v/v* of methanol and dichloromethane mixture at a total solids content of 50 mg/mL. The resulting ASD powders were vacuum-dried overnight, cryo-milled, and sieved to obtain the desired particle size of 106–250 μm. ASDs produced in this manner were confirmed to be crystal-free using polarized light microscopy (PLM) and powder X-ray diffraction (PXRD).

#### 2.2.4. Measurement of Glass Transition Temperature by Differential Scanning Calorimetry (DSC)

Glass transition temperature (T_g_) analysis of neat amorphous drug, polymer, and ternary ASD powders was performed using a TA Q2000 DSC with a refrigerated cooling accessory (TA Instrument, New Castle, DE, USA). Ternary ASD powders at a single drug loading (30% DL) but varied TPGS levels were measured to understand the impact of surfactant on the T_g_. Approximately 3–5 mg of the sample was equilibrated at 0 °C and heated to 155 °C at 5 °C/min with modulation of 1 °C every 60 s and then cooled back down to 0 °C followed by a second heating cycle with a 5 °C/min ramp and modulation of 1 °C every 60 s. The second heating cycle was used for T_g_ analysis. A constant 50 mL/min nitrogen gas flow rate was used during the run. Temperature calibration of the DSC was performed using indium at various heating ramps, including 5 °C/min.

#### 2.2.5. Surface Normalized Dissolution Rate Experiments

Surface normalized dissolution rates for drug, polymer, and surfactant from ternary ASDs (2%, 5%, and 10% TPGS) were determined using a Wood’s intrinsic dissolution rate assembly (Agilent Technologies, Santa Clara, CA, USA). It is assumed that the dissolving surface area remains constant throughout the experiment and a renewed layer of compact is exposed to the dissolution medium periodically. First, 100 mg of the ternary ASD powder was tableted in an 8 mm die (surface area of 0.5 cm^2^) using a Carver hydraulic tablet press at a force of 1500 psi held for a minute. The die containing the tablet was immersed in 100 mL dissolution medium and stirred at a rate of 100 rpm. Then, a 2 mL sample was aliquoted at the desired time points and replaced with fresh buffer. Out of the 2 mL sample, a 100 µL aliquot was used as is for drug and surfactant analysis, and the remaining portion was filtered through a nylon membrane filter (0.2 μm) and used for polymer analysis, after discarding the initial 1 mL of the filtrate. Methods for drug and polymer analysis were as described previously [4]. For TPGS quantification, a Waters XTerra RP C-18 column (150 mm × 4.6 mm, 3.5 μm particle size) was used and maintained at 40 °C. The mobile phase consisted of 90% acetonitrile and 10% methanol by volume. The flow rate was kept constant at 1.5 mL/min. The injection volume was 20 μL, and an ultraviolet (UV) detection wavelength of 284 nm was used. An Agilent 1260 infinity series HPLC (Agilent Technologies, Santa Clara, CA, USA) was used for all the analyses, and sample dilutions were done to match the organic to aqueous ratio of the mobile phase. A linear regression analysis was applied to the initial linear portion of the percent release versus time plot to calculate the surface-normalized dissolution rate of drug, polymer, and surfactant. The reference intrinsic dissolution rates of neat amorphous drug and polymer were taken as determined previously [4]. Two additional control experiments were also performed. In one experiment, the intrinsic dissolution rate of PVPVA containing 5% TPGS (no drug added), prepared by dissolving both components in a 1:1 methanol:dichloromethane mixture followed by rotary evaporation, was measured. In another experiment, drug and polymer release rates from 30% DL binary ASD were measured in the dissolution medium containing a pre-dissolved TPGS amount equivalent to that of 30% DL ternary ASD at 5% TPGS level, assuming 100% release (Table S2).

#### 2.2.6. Nanoparticle Tracking Analysis (NTA)

One mL of sample was withdrawn from the dissolution vessel at the end of dissolution experiments on various drug loading ternary ASDs at 5% TPGS level and nanoparticle tracking analysis (NTA) was performed using a NanoSight LM-10 (Malvern Instruments Inc., Westborough, MA, USA). NTA was also used to evaluate samples removed after surface normalized dissolution of 10% and 20% DL felodipine-PVPVA ASDs without TPGS. The samples were injected into the flow through cell of the instrument controlled at 37 °C and visualized via a 20× magnification objective after illuminating the sample with a green laser (75 mW, 532 nm) as a light source.

#### 2.2.7. Particle Size Distribution Using Dynamic Light Scattering (DLS)

The particle size of amorphous nanoparticles generated upon dissolution was evaluated by dynamic light scattering experiments performed using a Nano-Zetasizer (Nano-ZS) by Malvern Instruments (Westborough, MA, USA). A 173° back scattering detector was used for the measurements and 1 cm path length disposable plastic cuvettes were used as sample holders. Measurements were taken at 37 °C to maintain the sample at the dissolution temperature. The sampling approach was similar to the NTA measurements, and samples were withdrawn at the end of dissolution experiments for 5% TPGS ternary ASDs and 10% and 20% DL binary ASDs.

#### 2.2.8. X-ray Microcomputed Tomography (micro-CT) Images

X-ray microcomputed tomography (micro-CT) imaging was utilized to study the change in tablet morphology after partial dissolution (10 min timepoint), specifically for 30% DL ASDs as a function of TPGS level. Micro-CT images were collected using a high-resolution SkyScan-1272 microCT scanner (Bruker, Billerica, MA, USA). A spatial resolution of 3 μm/pixel was used, and two-dimensional scans were collected over a 180° rotation with steps of 0.1°. The X-ray tube was set to a current of 166 µm and a voltage of 60 kV. The partially dissolved tablet was vacuum dried overnight before imaging. Image reconstruction was performed using NRecon software (Bruker, Billerica, MA, USA, version 1.6.9.8) and visualized using *Dataviewer* software (Bruker, Billerica, MA, USA).

#### 2.2.9. ASD Film Exposure Studies under a High RH Environment

**Preparation of ASD films.** Felodipine-PVPVA ASD films at 30% DL and various TPGS levels (0%, 2%, 5%, and 10% by weight) were prepared using a KW-4A spin coater (Chemat Technologies Inc., Northridge, CA, USA) placed in a glove box under a nitrogen purge. An RH of less than 20% was ensured during film preparation to avoid water vapor-induced phase separation [31], and ambient temperature conditions (25 ± 2 °C) were used. ASD components were dissolved at the desired ratio in a 50:50 *v/v* methanol:dichloromethane mixture at a solids loading of 50 mg/mL followed by spin coating. A 20 µL solution was spin-coated using a two-step spin coating procedure, 500 rpm for 6 s and 3000 rpm for 30 s, on a ZnS substrate (SM-nIR2-Flat-5, Anasys Instruments, Santa Barabara, CA, USA). Then, ASD films were vacuum dried overnight before using them for high RH exposure studies.

**High RH exposure studies.** In order to study the impact of a water-saturated environment on the phase behavior of initially miscible ASD films, ASD films were exposed to ≈100% RH for 12 h using a chamber saturated with water vapor. After exposure, films were vacuum-dried overnight and then analyzed using atomic force microscopy for topographical and nano-scale infrared (nano-IR) imaging.

**Atomic force microscopy (AFM) topographical imaging.** An Anasys nanoIR2 instrument (Anasys Instruments, Santa Barbara, CA, USA) was used to perform AFM topographical imaging. A contact mode probe (model: PR-EX-NIR2, Anasys Instruments, Santa Barbara, CA, USA) was used to collect images. A 10 μm × 10 μm scan area was imaged at a scan rate of 0.5 Hz with an x and y resolution of 256 points. Analysis studio software (Anasys Instruments, Santa Barbara, CA, USA) was used for image collection.

**Bulk infrared (IR) spectroscopy.** To collect the reference IR spectra of neat amorphous drug and polymer, thin films of felodipine and PVPVA were spin coated onto KRS-5 substrates using the spin-coating procedure as described above. A Bruker Vertex 70 FTIR spectrophotometer (Bruker, Billerica, MA, USA) was used to collect spectra in transmission mode. A total of 128 scans were co-averaged over a spectral range of 1200–1800 cm^−1^ at a resolution of 4 cm^−1^. OPUS software (version 7.2, Bruker, Billerica, MA, USA) was used for data collection and analysis.

**NanoIR imaging.** NanoIR imaging was also done using the aforementioned Anasys nanoIR2 instrument, whereby AFM combined with a tunable IR laser source provides spatial resolution in the sub-micrometer range for IR imaging. The topographical imaging contact mode probes were also used for nanoIR imaging. The sample was irradiated by an IR laser at a wavelength corresponding to the sample’s absorption bands (1213 cm^−1^ for felodipine and 1243 cm^−1^ for PVPVA), which resulted in thermal expansion at the irradiation spot, leading to oscillation of the cantilever probe in contact with the sample. The characteristic “ringdown” amplitude of the cantilever oscillations recorded by the software at varying locations of the sample is proportional to the localized absorption of the sample. This helps in generating a chemically differentiating IR image as a complement to the AFM topographical image. The second harmonic mode of cantilever oscillation at a frequency of 200 kHz with a tolerated width of 50 kHz was used in this case. The IR laser was operated at 0.5 mW power and the scan rate was 0.1 Hz. A total of 8 scans were co-averaged for a single IR image. The x and y resolution values were set at 256 points for both. Ratio images were constructed by dividing the IR images obtained at 1213 cm^−1^ by those obtained at 1243 cm^−1^.

## 3. Results

### 3.1. Measurement of TPGS CMC

TPGS CMC (in the absence and presence of polymer) was determined from solubility measurements as well as using the pyrene fluorescence method with results shown in Figures S1 and S2. The CMC of TPGS (in the absence of polymer) in 100 mM pH 6.8 phosphate buffer at 37 °C was found to be approximately 50 μg/mL (Table 1), which is in agreement with previous reports [32]. The CMC in the presence of polymer (900 μg/mL PVPVA), also referred to as the critical aggregate concentration (CAC), was found to be approximately 40 μg/mL (Table 1). Nine hundred μg/mL was chosen as a representative PVPVA concentration to study the impact of drug–polymer interaction, if any, on the process of micelle formation. There was good agreement between the CMC/CAC values obtained by the two different methods employed. Only a minimal difference was found between CMC (without polymer) and CAC (with polymer) values, whereby this difference is insignificant in terms of the TPGS concentrations used in this study (assuming complete release), which are both above and below CMC values depending on the drug loading and % TPGS in the formulation (Table S2). This suggests that there is little-to-no interaction between TPGS and PVPVA that impacts the CMC. Furthermore, the crystalline solubility of felodipine in the presence of monomeric TPGS was similar to that in buffer alone (≈1.3 μg/mL) [4], indicating that no significant solubilization of felodipine occurs in the absence of TPGS micelles.

### 3.2. Amorphous Solubility

The amorphous solubility of felodipine in the presence of 900 μg/mL PVPVA was found to be 9.5 ± 1.1 (*n* = 3, mean ± SD) µg/mL, which is within the experimental error of the previously reported value in the absence of polymer (8 µg/mL) [4]. Since no solubilization was observed for crystalline felodipine in the presence of monomeric TPGS (Figure S1), it is assumed that TPGS does not cause a change in the amorphous solubility of felodipine below its CMC.

### 3.3. Glass Transition Temperature (T_g_)

The measured glass transition temperatures (inflection point on DSC thermogram) of amorphous felodipine, PVPVA, and 30% DL ASDs at various TPGS levels (0%, 2%, 5%, and 10%) are presented in Table 2. All ASDs showed a single glass transition temperature with no melting endotherm, which is consistent with a single phase ASD. In comparison to the binary ASD, the inclusion of TPGS resulted in reduced T_g_ in the ternary ASDs.

### 3.4. Surface-Normalized Release Profiles of Drug, Polymer, and Surfactant from Ternary ASDs

**Figure 2:**
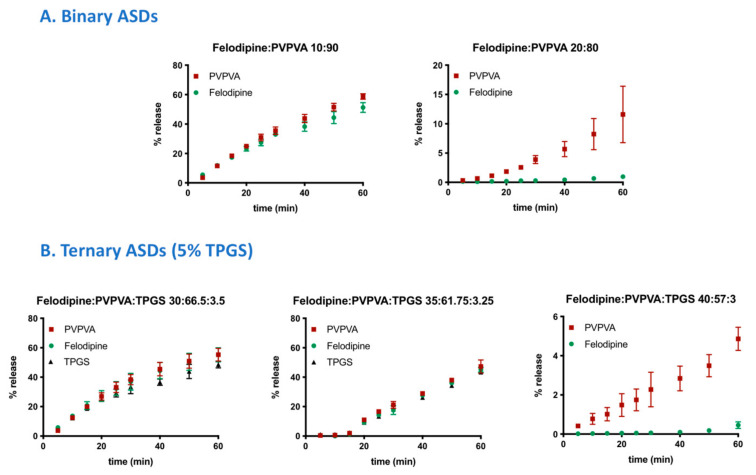
Comparison of the release rates of drug, polymer, and TPGS (where applicable) at drug loadings where a switch between polymer-controlled and drug-controlled release is observed. (**A**) Binary ASDs [4] and (**B**) ternary ASDs (with 5% TPGS). The ratios in the legend represent weight ratio of components in the ASDs. Error bars represent standard deviations, *n* = 3, and they may be smaller than symbols in some instances. TPGS levels were below the quantification limit for 40% DL ternary ASDs.

**Figure 3:**
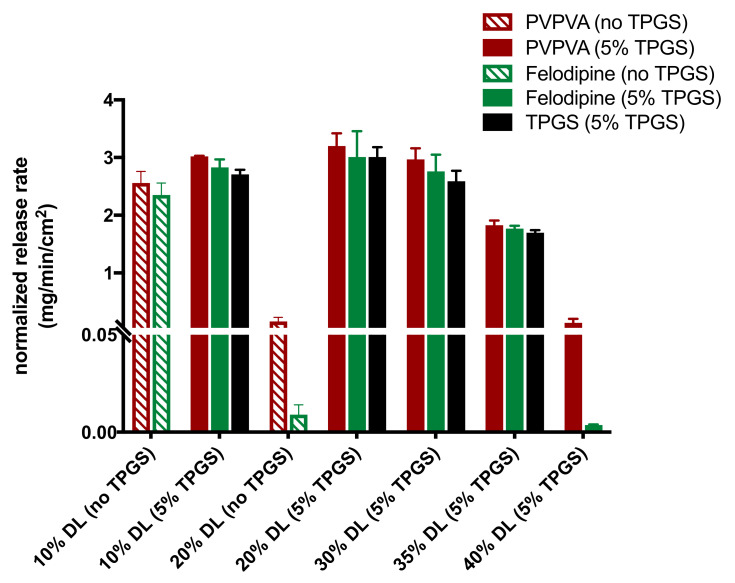
Surface-normalized dissolution rates for amorphous felodipine, PVPVA, and TPGS (where applicable) for different drug loading ASDs without TPGS [4] and with 5% TPGS. Error bars represent standard deviations, *n* = 3. TPGS levels were below the quantification limit for 40% DL ternary ASDs.

**Figure 4:**
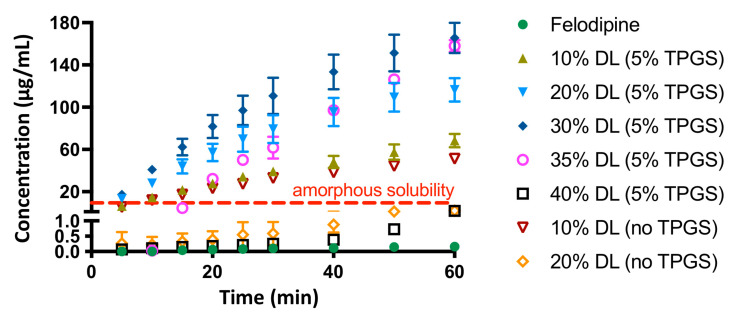
Concentration versus time profile for felodipine from amorphous drug alone, and as part of a PVPVA-based ASD with and without 5% TPGS. The legends represent drug loadings (by weight) and TPGS% in the ASDs. Error bars represent standard deviations, *n* = 3, and may be smaller than symbols in some instances.

Reference release profiles of binary ASDs of felodipine and PVPVA (10% and 20% DL), from a previous study, are shown in Figure 2A [4]. The release profiles of drug, polymer, and surfactant from ternary ASDs containing 5% TPGS where a switch between polymer-controlled and drug-controlled release is observed are shown in Figure 2B. A clear contrast between the drug loading dependent dissolution behavior of felodipine-PVPVA ASDs with and without TPGS can be seen. The drug loading “cut-off” up to which drug and polymer release congruently (limit of congruency (LoC)) is between 10% and 20% DL for ASDs without TPGS [4]. However, with 5% TPGS, the LoC increases to 35% DL. To compare the release rates of each ASD component, the surface-normalized dissolution rates of drug, polymer, and surfactant as a function of drug loading, and with and without TPGS are summarized in Figure 3. The difference in the drug release rate between congruent (polymer-controlled) versus incongruent (drug-controlled) release behavior stems from the orders of magnitude difference between the neat polymer release rate (PVPVA release rate: ≈3 mg/min/cm^2^) and neat amorphous drug release rate (amorphous felodipine release rate: ≈0.001 mg/min/cm^2^) [4]. Notably, felodipine concentrations upon the dissolution of congruently releasing ASDs exceed the amorphous solubility, while incongruently releasing ASDs show negligible amounts of drug release, and drug concentrations remain below amorphous solubility, as shown in Figure 4. Clearly, the presence of TPGS significantly improves the drug loading range up to which polymer-controlled release can be achieved.

**Figure 5:**
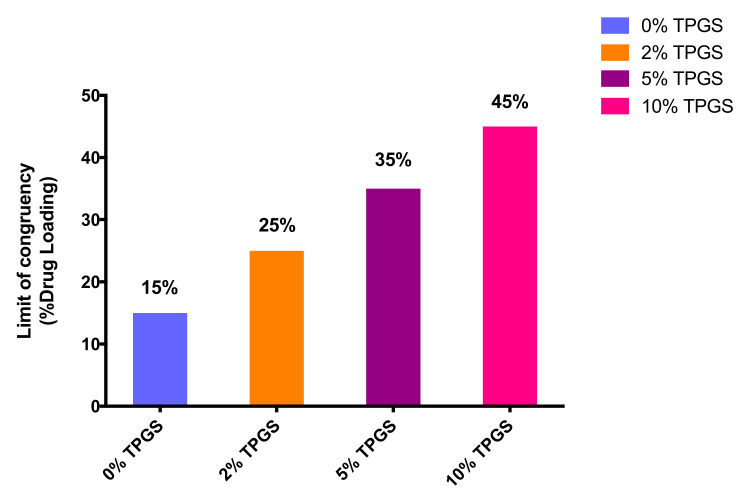
Limit of congruency (% drug loading by weight) for felodipine:PVPVA ASDs at different levels (0% [4], 2%, 5%, and 10% TPGS).

**Figure 6:**
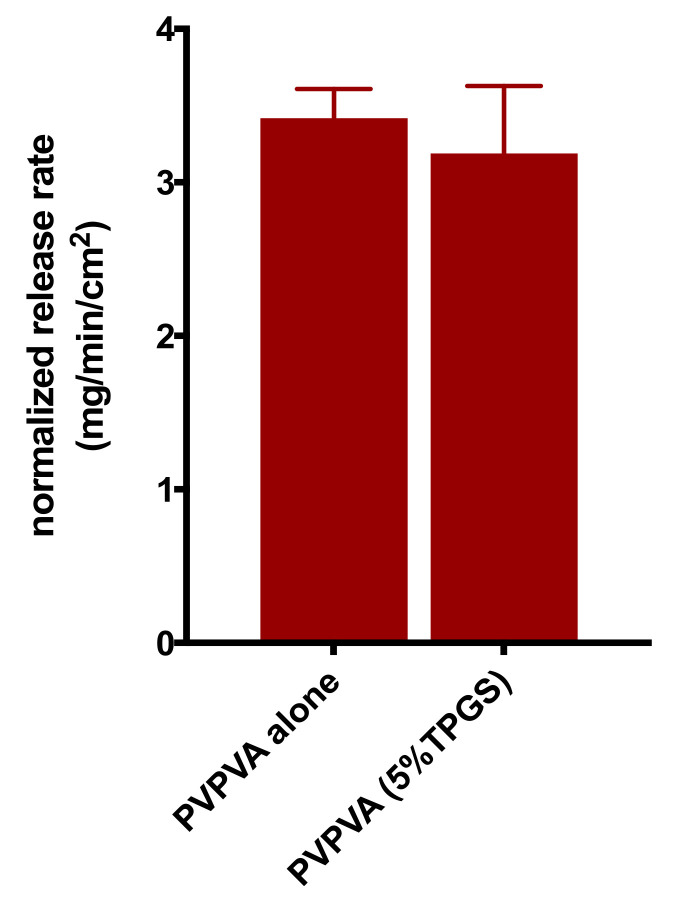
Surface-normalized dissolution rates for PVPVA alone [4] and PVPVA with 5% TPGS. Error bars represent standard deviations, *n* = 3.

**Figure 7:**
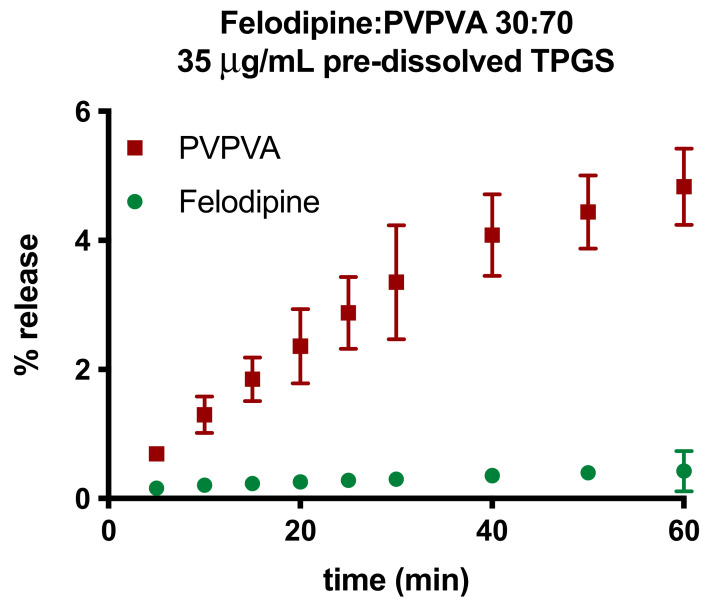
Percent release versus time profiles for amorphous felodipine and PVPVA for 30% DL binary ASD (without TPGS) with pre-dissolved TPGS in the dissolution medium. The ratio in the legend represents weight ratios of ASD components and the amount of pre-dissolved TPGS (35 μg/mL). Error bars represent standard deviations, *n* = 3, and they may be smaller than symbols in some instances.

Surface-normalized dissolution rates for drug and polymer for different drug loading ASDs with 2% and 10% TPGS were also determined (Figure S3). The LoC was found to increase with TPGS level, as shown in Figure 5. Additional control experiments were also performed to understand the role of TPGS in improving felodipine release at higher drug loadings. PVPVA dissolution in the presence of 5% TPGS did not increase relative to the surface-normalized release rate for neat PVPVA (Figure 6) [4]. Furthermore, TPGS pre-dissolved in the dissolution medium (at a concentration equivalent to a 30% DL ternary ASD with 5% TPGS, assuming 100% release) did not improve the drug-release rate (Figure 7), indicating that it is important to have the surfactant as part of the ASD matrix to realize a release advantage.

### 3.5. Nanoparticle Tracking Analysis (NTA)

**Figure 8:**
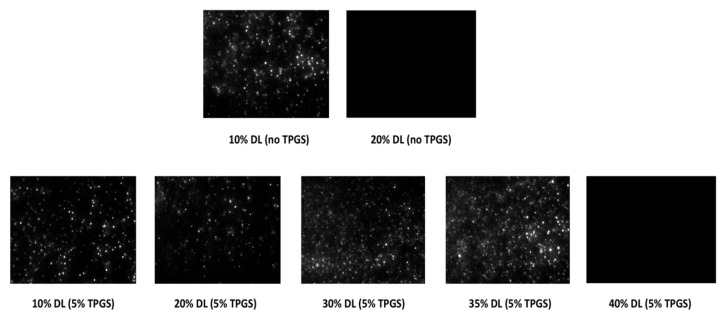
NTA scattering images of solutions obtained after dissolution of felodipine-PVPVA ASDs without TPGS (upper panel) and with 5% TPGS (lower panel). The bright spots indicate the presence of light scattering species where the field of view is 100 by 80 μm.

Figure 8 shows the representative NTA images acquired on solutions removed after 60 min of surface normalized release experiments. NTA provides visual evidence of amorphous nanoparticles by detecting the light scattered as they move under Brownian motion. It was observed that ASDs with drug loadings up to the LoC, i.e., 10–35% DL with 5% TPGS, and 10% DL without TPGS, led to the formation of amorphous nanoparticles upon dissolution.

### 3.6. Dynamic Light Scattering (DLS)

**Figure 9:**
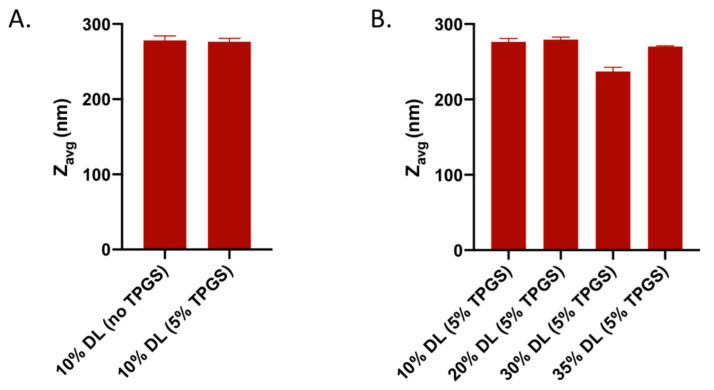
Z-average (Z_avg_) particle diameter for amorphous nanoparticles generated upon dissolution: without and with TPGS (5%) at 10% DL (**A**) and at different DLs with 5% TPGS level (**B**).

The average particle size diameters of amorphous nanoparticles at the end of dissolution experiments (60 min timepoint) of select ASDs are presented in Figure 9. Once released, nanoparticle sizes remained relatively stable over the 60 min time frame, and no signs of agglomeration were observed (data not shown); therefore, for the sake of comparison, only the 60 min timepoint data are shown. The size of the amorphous nanoparticles generated upon dissolution was in the range of 200–300 nm with a unimodal distribution across different ASDs (Figure S4), with no specific trend being observed as a function of drug loading, for different DLs studied at the 5% TPGS level, or with respect to TPGS presence or absence, for 10% DL ASDs. It should be noted that our observations here with respect to felodipine amorphous nanoparticle size trend with TPGS level is different from that of a previous study with TPGS involving another API, anacetrapib [14]. The authors in the aforementioned study found that the nanoparticle size in solution decreased as a function of increasing TPGS level in the ternary ASD at a similar drug loading.

### 3.7. X-ray Microcomputed Tomography (Micro-CT) Imaging

**Figure 10:**
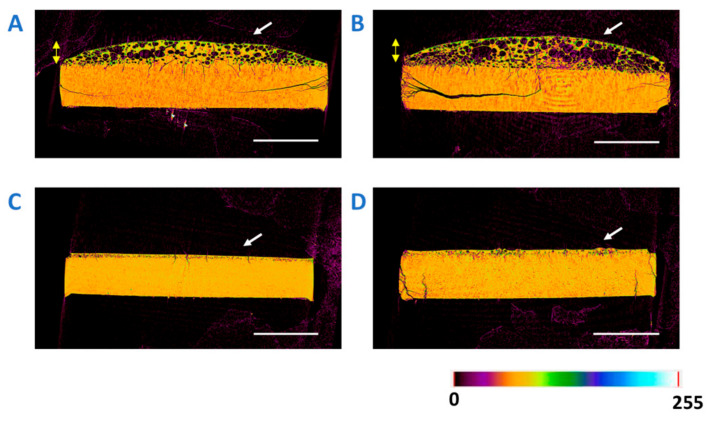
X-ray microcomputed tomography cross-sectional images for the 30% DL ASD tablets with 0% TPGS (**A**), 2% TPGS (**B**), 5% TPGS (**C**), and 10% TPGS (**D**) after 10 min of dissolution. Scale bar (in white) is 2 mm. Arrow (in white) on the images is pointing toward the dissolution front of the tablet. The color bar represents the range of density measurement from lowest (0) to highest (255).

Micro-CT imaging was employed to monitor the evolution of 30% DL ASD tablet surface morphology as a result of partial dissolution (10 min timepoint) for various TPGS levels, as shown in the images displayed in Figure 10. A clear difference in surface morphologies of the partially dissolved tablet front was observed at lower (0% and 2%) versus higher (5% and 10%) TPGS levels. While the tablets with no or 2% TPGS showed a porous surface layer, tablets with 5% and 10% TPGS had a relatively homogeneous morphology throughout the tablet thickness. The evolution of a porous microstructure is consistent with the release behavior of low TPGS level (0% and 2%) ASDs, whereby polymer dissolves faster than the drug, leaving behind a porous drug-rich interface on the tablet surface, which is a phenomenon previously observed for other systems with incongruent release patterns [4,7].

### 3.8. Phase Behavior of ASD Films after High RH Exposure

**Figure 11:**
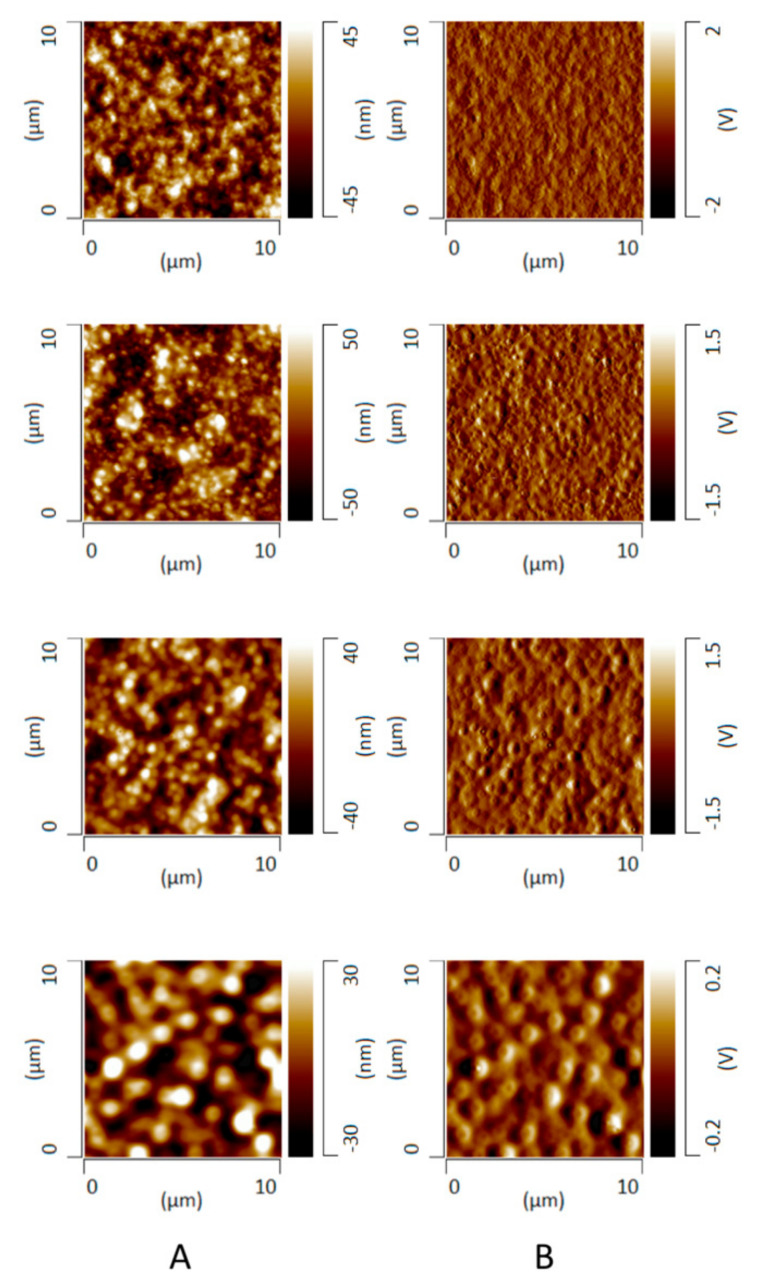
AFM topographical (**A**) and deflection images (**B**) obtained after incubation of 30% DL felodipine-PVPVA ASD films in a water-saturated chamber for 12 h. The films contained various levels of TPGS: 0%, 2%, 5%, and 10% (from top to bottom).

Atomic force microscopy (AFM) topographical imaging was performed in order to study the differences in the microstructure evolution of initially miscible 30% DL ASD films stored in a water-saturated environment as a function of surfactant level. Figure 11 shows the AFM topographical images representing variations in sample height alongside corresponding deflection images representing variations in cantilever deflection during the scan. Both image types suggested inhomogeneous or phase-separated ASD films after high RH exposure, albeit with gradients in the variations of the resultant microstructure as a function of TPGS level. For ASDs containing no, or a low level of TPGS content (0% and 2%), irregularly shaped semi-continuous domains were formed. In contrast, for ASDs with higher TPGS content (5% and 10%), circular, discrete domains were observed. There was no significant difference in the domain sizes in the z direction between ASD films with different TPGS levels, which was indicated by the comparable height variations of the AFM images. It should be noted that all of the freshly prepared ASD films were confirmed to be homogeneous and were devoid of any phase-separated features (Figure S5).

**Figure 12:**
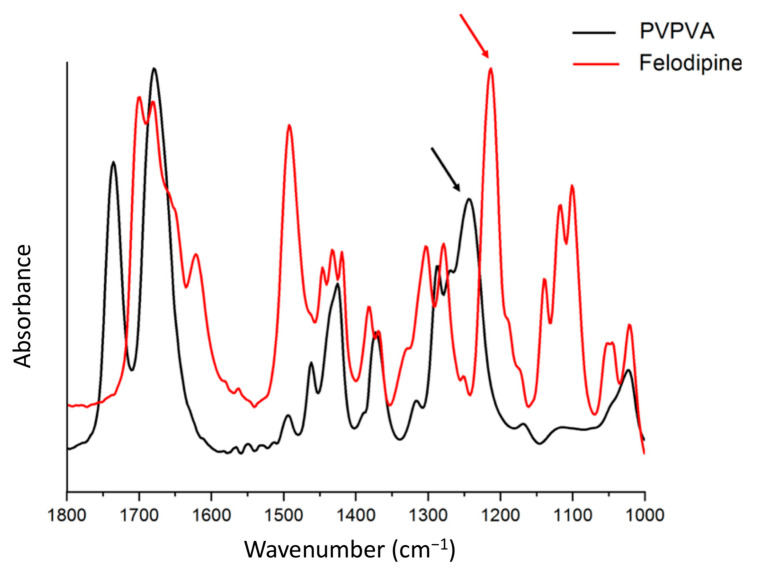
Bulk IR spectra of amorphous felodipine and PVPVA. The amorphous felodipine peak at 1213 cm^−1^ (indicated by red arrow) and PVPVA peak at 1243 cm^−1^ (indicated by black arrow) were used to differentiate drug-rich and polymer-rich phases in phase-separated ASD films.

**Figure 13:**
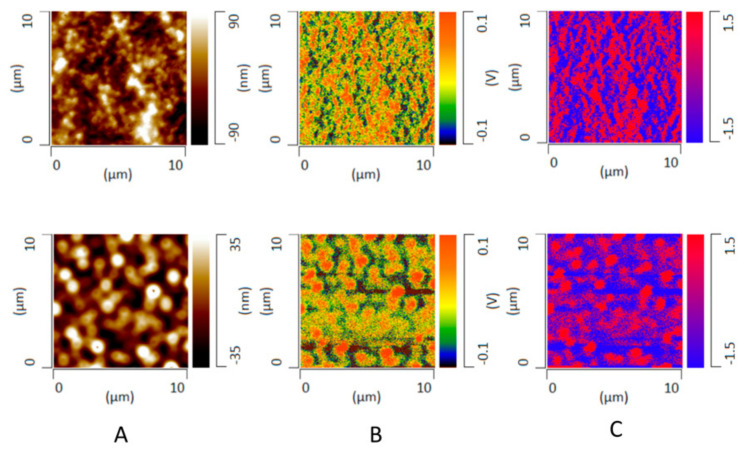
AFM nanoIR images of 30% DL felodipine-PVPVA ASD films, without TPGS (top panel) and with 10% TPGS (bottom panel), after storing in a water-saturated chamber for 12 h: (**A**) topographical image, (**B**) IR image at 1213 cm^−1^, and (**C**) IR ratio image (1213 cm^−1^/1243 cm^−1^), where red areas are drug-rich.

Bulk infrared (IR) spectra of amorphous felodipine and PVPVA films were collected in transmission mode as shown in Figure 12. IR peaks at 1213 cm^−1^ and 1243 cm^−1^ were identified to be chemically discriminating for drug and polymer, respectively. To confirm the phase separation and identify the drug-rich and polymer-rich phases, AFM nano-IR imaging in conjunction with AFM topographical imaging was performed on ASD films without TPGS and at a 10% TPGS level after 12 h of exposure to a water-saturated environment. The topographical image, IR image at 1213 cm^−1^ (felodipine peak), and ratio image of the 1213 cm^−1^ peak over the 1243 cm^−1^ peak for both systems are shown in Figure 13. The IR images show good agreement with the AFM topographical images, confirming the phase separation of initially miscible films upon exposure to high relative humidity conditions, whereby elevated height features were confirmed to be drug-rich, and the continuous phase surrounding them was confirmed to be polymer-rich or drug-lean. A clear contrast was seen between microstructures of phase separated ASD films with and without TPGS, where the contiguous nature of drug-rich domains was evident for the 30% DL binary ASD film with no TPGS, and spherical, well-segregated domains were characteristic of the 30% DL ternary ASD film.

## 4. Discussion

The pill burden is a major challenge in the design of amorphous solid dispersion (ASD)-based formulations. Beyond physical stability constraints, a decline in drug release rate with an increased drug loading requires a high polymer/drug ratio, which is a major contributor toward the increased pill burden of these enabled formulations. There is emerging evidence that the decline in release performance is concomitantly associated with a change in dissolution mechanism, from “polymer-controlled” or congruent release of ASD components at low drug loadings to “drug-controlled” or incongruent release at higher drug loadings [4,7,8,16,33,34]. It should be noted that most of these studies focus on PVPVA-based ASDs, and the generality of these observations to ASDs formulated with other polymers is not fully established. The limit of congruency (LoC), which is the drug loading boundary above which the dissolution mechanism switches from being congruent to incongruent, varies from one drug-PVPVA system to another; however, it typically occurs at <25% drug loading and often acts as a limiting factor in achieving reasonable dosage sizes, especially for high dose drugs. In order to develop strategies to overcome this limitation, researchers have pursued understanding of this sudden change in dissolution mechanism at the LoC, whereby changes in the ASD microstructure and homogeneity during hydration appear to be important to the generation of a drug-rich layer at the surface of the ASD, which in turn results in slow drug release [4,7,14,17,35,36,37]. Once the water enters the ASD, it acts as an anti-solvent for the drug, generating a thermodynamic driving force for phase separation. This phenomenon has also been described by the delta chi effect [38,39], which describes the different affinity of water for the drug and polymer components of the ASD. Furthermore, since water also acts as a plasticizer [40], it enhances molecular mobility in the ASD, facilitating any thermodynamically favored phase separation processes. The evolution of microstructure as a result of phase separation, in particular, domain shape, size, and composition, has been found to be consequential in influencing the dissolution mechanism at a particular drug loading for a given drug–polymer system and is possibly the reason underlying the conflicting reports of the impact of phase separation on drug release from ASDs found in the literature [7,14,35,41,42]. Upon taking stock of the limited published literature with direct supporting evidence for the impact of ASD microstructure on dissolution performance, we have found evidence that cases where ASDs either show no evidence of water-induced phase separation, or where ASDs phase separated into discrete drug-rich domains of sub-micron sizes, were concomitantly associated with a polymer-controlled (congruent) release mechanism and exhibited faster drug release rates. On the other hand, when the hydrated ASDs showed semi-continuous or continuous drug-rich phases, the drug release mechanism was rendered incongruent with faster polymer release and a much slower drug release rate. For example, in a study by Han et al. [36], for ritonavir–polyvinylpyrrolidone ASDs, the drug loading level at which the dissolution mechanism changed from being congruent to incongruent coincided with a change in phase separation morphology, whereby hydrophobic drug-rich domains were discrete in the former case, while a drug-rich continuous phase was found in the latter case. Similar, in the lopinavir–hydroxypropylmethylcellulose (HPMC) ASD film “immersion” study by Li and Taylor [35], only the lowest drug loading studied, 15% DL, showed discrete domains followed by complete dissolution, whereas apparent contiguous drug-rich material was left behind on the substrate for higher drug loadings as a result of incomplete dissolution. It should be noted that the authors did not measure polymer dissolution in this work but made the case for creating microstructure in ASDs with discrete drug-rich domains for improved dissolution performance due to co-release of drug-rich domains and polymer.

In a pharmaceutical drug product development environment, the drug’s physicochemical properties are dictated by the molecular features incorporated for maximizing target affinity. If the drug properties necessitate an enabling formulation route, polymer screening is then done using a handful of spray drying or hot melt extrusion-amenable, commercially available polymers. The polymer choice for an ASD is in large part typically based on its nucleation inhibition properties for the drug in the solid and solution states, which in turn is dependent on polymer glass transition temperature, polymer hygroscopicity, and drug–polymer interactions. More recently, the impact of polymer choice on the formation and stabilization of amorphous nanoparticles upon ASD dissolution has also started to gain attention [34,43]. Nevertheless, the limit of congruency is a property of a given drug–polymer ASD system, and it can be identified but not altered without changing the polymer. Therefore, an approach that results in the creation of a favorable microstructure in the high drug-loaded hydrated ASD matrix upon dissolution with the formation of discrete sub-micron drug-rich domains could potentially overcome the drug loading dependent dissolution limitation of ASDs. There are accounts in the literature where the addition of surfactant to the ASDs has resulted in improved dissolution performance as a function of drug loading, although the associated mechanisms have not been deciphered to date [16,44]. Harmon et al. evaluated the release performance of ternary ASDs with surfactant but only at a single drug loading [14]. Herein, we have studied drug and polymer release from ternary ASDs as a function of drug loading at varied surfactant levels, clearly demonstrating an improved LoC, which was a function of surfactant level. Thus, it is pertinent to consider the likely underlying reasons for the observed improvements in release resulting from surfactant incorporation.

### 4.1. Solubilization

Surfactants are well known to enhance drug solubility through micellar solubilization [45,46], and thus, this is an obvious factor to consider. For conditions used in this study, TPGS CMC ranges between 40 and 50 µg/mL, whereby the final TPGS concentration in the bulk medium (assuming 100% release) is both below and above CMC depending on drug loading and surfactant level (Table S2). We observed no correlation between the presence or absence of micellar TPGS and the resultant release behavior. For example, the LoC for felodipine-PVPVA ASDs increases from 15% DL without TPGS to 25% DL with 2% TPGS, although final TPGS concentrations remain below the CMC (Figure 5, Table S2). Similarly, for 30% DL, TPGS level is below CMC for a 5% TPGS ASD and above CMC for the 10% TPGS ASD (Table S2); however, both ASDs show a congruent release of components (Figure 3 and Figure S3). Thus, solution solubilization by TPGS micelles plays a negligible role in impacting the LoC. This was further corroborated with a control experiment where TPGS was pre-dissolved in the dissolution medium at an equivalent level to the 30% DL ternary ASD with 5% TPGS. While the 30% DL ternary ASD showed congruent release (Figure 2 and Figure 3), the corresponding 30% DL binary ASD still showed incongruent release of drug and polymer (Figure 7), despite the presence of the pre-dissolved surfactant. These observations indicate that surfactants impact release beyond any benefit incurred from wetting improvement, and they point to the importance of incorporating the surfactant as an ASD component rather than as an external excipient in the formulation. Furthermore, these results suggest that the improvement in LoC in the presence of the surfactant is a phenomenon associated with the hydrated matrix or the interface between the ASD matrix and the bulk solution.

### 4.2. Hydrated ASD Microstructure and Dissolution

**Figure 14:**
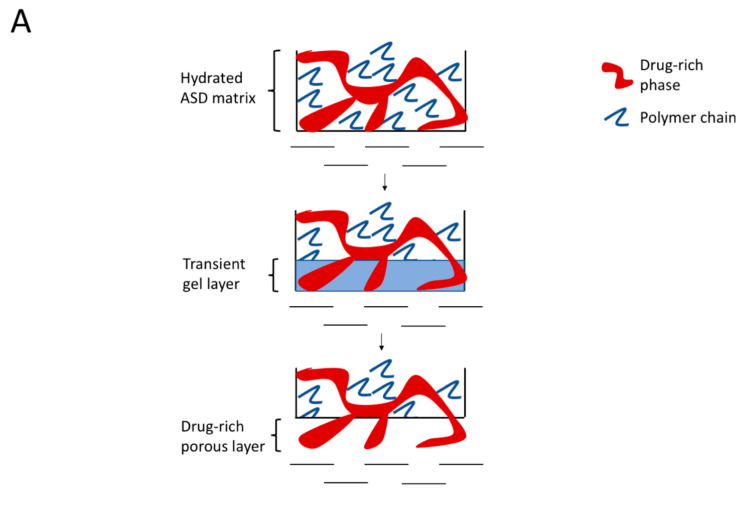

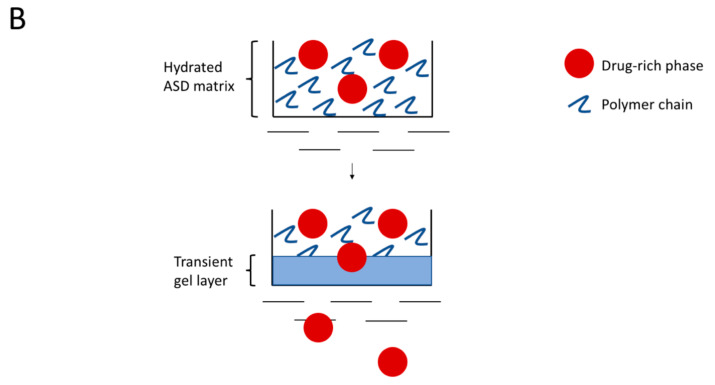
Schematic showing microstructure evolution and dissolution process of drug and polymer from high drug-loaded ASDs at lower and higher surfactant levels: (**A**) Phase behavior and dissolution process of 30% DL felodipine-PVPVA ASDs without TPGS and 2% TPGS, whereby phase separated drug-rich and polymer-rich phases release incongruently, leaving behind a porous drug-rich layer on the tablet surface and (**B**) Phase behavior and dissolution process of 30% DL felodipine-PVPVA ASDs with 5% and 10% TPGS, whereby drug-rich domains release intact as the polymer dissolves and the transient gel layer recedes, leading to the congruent release of drug and polymer (Adapted with permission from [48], Elsevier, 2002).

Distinct differences in morphology were seen following exposure to water depending on the presence or absence of surfactants, both in films where no mass transfer occurred, and at the tablet interface, following partial dissolution. Based on these microstructural changes and the release behavior observed for the 30% DL ASDs, we propose a release model as outlined in the schematic shown in Figure 14. The model is an extension of our previous model proposed for binary ASD systems [17], and it works on the premise of the presence of a “transient gel layer” at the dissolving interface of the ASD tablets, which is a typical behavior for amorphous polymers [47]. The transient gel layer here refers to an interface between the dry ASD matrix and the aqueous phase, where the polymer chains are plasticized by solvent ingress. The polymer chains disentangle from the gel layer prior to releasing into the dissolution medium. We propose that the ASD dissolution mechanism at high drug loadings, whether drug-controlled or polymer-controlled, depends largely upon the “architecture” of the drug-rich phase that evolves as a result of phase separation in the hydrated ASD matrix, assuming that phase separation occurs faster than polymer dissolution. Felodipine-PVPVA, 30% DL ASDs without TPGS or at a low TPGS level (2%) show a contiguous drug-rich phase forming a “network-like” structure as depicted in Figure 14A and observed in AFM images of Figure 11 and Figure 13. This contiguous structure likely also forms in the transient gel layer during dissolution forming a porous, insoluble layer on the ASD tablet surface, where the pores are generated by release of the polymer-rich phase (Figure 10A,B). This leads to incongruent release due to the differential solubility of the drug-rich and the polymer-rich phases. In contrast, ASDs containing higher TPGS levels (5% and 10%) show persistent discrete drug-rich domains embedded in the continuous polymer-rich phase as depicted in Figure 14B and observed in the AFM images of Figure 11 and Figure 13. The difference in morphology of the phase-separated regions can be rationalized based on the reduction in the interfacial energy between the two phases in the presence of TPGS, which in turn will reduce the coarsening rate, enabling discrete drug-rich domains to persist. These discrete drug-rich domains can release as intact domains when the transient gel layer recedes upon polymer dissolution, as shown in Figure 14B. Alternatively, the presence of the surfactant may reduce the kinetics of phase separation; if phase separation kinetics in the gel layer are slower than polymer dissolution, then congruent release is anticipated. It is important to note that the AFM images provide a snapshot of phase separation following 12 h of equilibration at 100% RH, which is a very different timescale from that experienced by the dissolving compact.

### 4.3. Molecular Mobility

Surfactant acts as a plasticizer, decreasing the glass transition temperature (T_g_) of ternary felodipine-PVPVA ASDs (Table 2) and increasing molecular mobility of the ASD components. A concomitant mechanism for the observed increase in the LoC of felodipine-PVPVA ASDs could be related to this increase in molecular mobility. Polymer disentanglement only commences when enough solvent has entered the matrix to sufficiently plasticize the system [47,49]. A lowered T_g_ should theoretically enable polymer disentanglement to commence at an earlier time point when less water has permeated into the matrix, favoring polymer dissolution. Consequently, enhanced polymer dissolution in the presence of drug and surfactant may compete favorably against the kinetics of phase separation, preventing the formation of the drug-rich surface layer responsible for drug-controlled release at higher drug loadings. Thus, both the enhanced molecular mobility and/or favorable morphology of phase-separated drug-rich domains in the hydrated ASDs could help drive the higher LoC of ternary ASDs. The impact of T_g_ relative to dissolution temperature on ASD release has been observed previously. For example, indomethacin-PVPVA ASDs showed a reduced LoC when the experimental temperature was lowered to below the T_g_, highlighting that molecular mobility plays an important role [17].

### 4.4. Mechanism of Amorphous Nanoparticles Formation

The conflicting reports in the literature [7,14,35,41,42] with regard to the impact of solid-state amorphous phase separation, also known as amorphous–amorphous phase separation (AAPS), on ASD dissolution performance, can possibly be explained by considering the role of phase separation morphology and kinetics. The origin of the amorphous nanoparticles observed in the solution phase and the correlation, if any, with water-induced AAPS in the matrix, is still an unresolved issue. For example, Harmon et al. suggested that phase-separated amorphous drug-rich domains are formed within the hydrated ASD matrix and later are released as nanoparticles into the solution, which is driven by concomitant polymer diffusion into bulk solution [14]. Indulkar et al. performed a mechanistic isotope scrambling study and suggested an alternate mechanism, whereby the nanoparticles are generated as a result of phase separation of drug in excess of the amorphous solubility in the aqueous phase following ASD dissolution [13]. These studies were conducted with different model systems under different experimental conditions, and therefore, they do not necessarily present mutually exclusive mechanisms, especially given the complex dissolution of polymeric systems whereby multiple zones with varying levels of hydration can exist between the dry matrix core and the bulk aqueous medium [47]. In this study, by comparing the range of domain sizes formed on the ASD film surface as characterized by AFM (Figure 11 and Figure 13) and the consistent Z_avg_ values of nanoparticles in the solution (200–300 nm) as measured by DLS (Figure 9), it becomes clear that the domains generated in the ASD film are larger than the nanoparticles observed in solution. The lack of correlation between the drug-rich domains observed in the hydrated film and the solution suggests that the local environment at the time of formation and thereafter has a major impact on the resultant morphology. This is consistent with phase separation likely occurring in different regions of a phase diagram for films versus dissolution experiments, depending on the rate of quenching into an unstable region, which in this instance is driven by the increase in water content. Thus, the rate of quenching is expected to be faster for the dissolution environment (more rapid ingress of water into the compact), relative to the RH exposure study where water diffuses from the environment into the film. In addition, the phase-separated regions may undergo coarsening via different mechanisms and rates, which will again be influenced by local environment (mobility, composition etc.), with relevant mechanisms including Ostwald ripening and coalescence. The AFM experiments serve to demonstrate the susceptibility of the system to phase separation in the presence of water, as well as the tendency of the drug-rich regions to minimize their surface area by forming larger, contiguous domains. However, they do not provide information about the kinetics of phase separation and how this relates to the release of drug and polymer from the ASD–water interface. The microCT results are supportive of the formation of continuous drug-rich domains in the absence of surfactant, in agreement with the AFM data, so a rapid coarsening of the drug-rich phase separated domains is clearly a feasible explanation for the loss of release performance; in other words, both phase separation and the resultant phase separated morphology are important for release. Based on AFM images, surfactant is clearly effective at enabling discrete domains of the drug-rich phase to exist, suggesting that surfactant reduces coarsening kinetics. In the context of drug release from high DL ASDs when surfactant is incorporated, it is unclear if the surfactant decreases the rate of phase separation such that molecular drug is able to release, and then liquid–liquid phase separation occurs, or if nanoparticles are formed in the hydrated matrix and are prevented from ripening by the surfactant. Ultimately, analytical approaches that enable the evolving microstructure of the ASD–water interface to be captured are likely to be useful for resolving these outstanding questions.

## 5. Conclusions

A systematic study of drug loading dependent dissolution mechanisms of felodipine-PVPVA ASDs as a function of TPGS level is reported. The addition of TPGS alleviated the low limit of congruency and thus the low drug loading limitation of binary felodipine-PVPVA ASDs. The limit of congruency scaled with the TPGS level in the ASDs, increasing from 15% drug loading with no surfactant to 45% drug loading with the addition of 10% TPGS to the ASD matrix. Based on a comparison of the microstructural evolution in the presence and absence of surfactant, TPGS is thought to positively impact drug release by preventing the formation of a continuous drug-rich layer at the ASD compact–water interface. Increased molecular mobility in the presence of the surfactant may also play a role in improving the limit of congruency of ASDs with higher surfactant levels. The result of this study indicates that the addition of appropriate surfactants to an ASD formulation is a potential strategy to design high drug loading ASDs without loss in dissolution performance.

## Figures and Tables

**Table 1:** Critical micelle concentration (CMC) of TPGS in pH 6.8 phosphate buffer with and without PVPVA.

Method	CMC (µg/mL)	
	Buffer	Buffer + PVPVA
By fluorescence	≈50	≈40
By solubility measurement	49 ± 4	41 ± 3

**Table 2:** Glass transition temperature for amorphous felodipine, PVPVA, and 30% DL felodipine-PVPVA ASDs at different TPGS levels (0%, 2%, 5%, and 10%).

Material	Glass Transition Temperature (°C)
Felodipine	44.7 ± 1.8
30% DL ASDs	
0% TPGS	89.2 ± 1.7
2% TPGS	86.0 ± 0.5
5% TPGS	80.8 ± 0.8
10% TPGS	71.1 ± 0.8
PVPVA	105.4 ± 1.9

## Data Availability

Not applicable.

## Supplementary Materials

The following are available online at https://www.mdpi.com/article/10.3390/pharmaceutics13050735/s1, Table S1: Physicochemical and thermodynamic properties of felodipine, Table S2: ASD compositions studied/referenced in this work, Figure S1: CMC of TPGS as determined by solubility measurement, Figure S2: CMC of TPGS as determined by pyrene fluorescence method, Figure S3: Surface normalized dissolution rates of drug, polymer and TPGS for 2% and 10% TPGS containing ternary ASDs, Figure S4: Representative DLS data, Figure S5: Representative AFM topographical images of initially prepared ASDs.

## References

[1] Rodriguez-Aller M., Guillarme D., Veuthey J.-L., Gurny R. (2015). Strategies for formulating and delivering poorly water-soluble drugs. J. Drug Deliv. Sci. Technol..

[2] Benet L.Z. (2013). The role of BCS (biopharmaceutics classification system) and BDDCS (biopharmaceutics drug disposition classification system) in drug development. J. Pharm. Sci..

[3] Hancock B.C., Parks M. (2000). What is the true solubility advantage for amorphous pharmaceuticals?. Pharm. Res..

[4] Saboo S., Moseson D.E., Kestur U.S., Taylor L.S. (2020). Patterns of drug release as a function of drug loading from amorphous solid dispersions: A comparison of five different polymers. Eur. J. Pharm. Sci..

[5] Jermain S.V., Brough C., Williams R.O. (2018). Amorphous solid dispersions and nanocrystal technologies for poorly water-soluble drug delivery—An update. Int. J. Pharm..

[6] McKelvey C.A., Kesisoglou F. (2019). Enabling an HCV Treatment Revolution and the Frontiers of Solid Solution Formulation. J. Pharm. Sci..

[7] Saboo S., Mugheirbi N.A., Zemlyanov D.Y., Kestur U.S., Taylor L.S. (2019). Congruent release of drug and polymer: A “sweet spot” in the dissolution of amorphous solid dispersions. J. Control. Release.

[8] Indulkar A.S., Lou X., Zhang G.G.Z., Taylor L.S. (2019). Insights into the Dissolution Mechanism of Ritonavir-Copovidone Amorphous Solid Dispersions: Importance of Congruent Release for Enhanced Performance. Mol. Pharm..

[9] Ilevbare G.A., Taylor L.S. (2013). Liquid-Liquid Phase Separation in Highly Supersaturated Aqueous Solutions of Poorly Water-Soluble Drugs: Implications for Solubility Enhancing Formulations. Cryst. Growth Des..

[10] Kesisoglou F., Wang M., Galipeau K., Harmon P., Okoh G., Xu W. (2019). Effect of Amorphous Nanoparticle Size on Bioavailability of Anacetrapib in Dogs. J. Pharm. Sci..

[11] Wilson V., Lou X., Osterling D.J., Stolarik D.F., Jenkins G., Gao W., Zhang G.G.Z., Taylor L.S. (2018). Relationship between amorphous solid dispersion in vivo absorption and in vitro dissolution: Phase behavior during dissolution, speciation, and membrane mass transport. J. Control. Release.

[12] Jara M.O., Warnken Z.N., Williams R.O. (2020). Amorphous solid dispersions and the confounding effect of nanoparticles in in vitro dissolution and in vivo testing: Niclosamide as a case study. bioRxiv.

[13] Indulkar A.S., Waters J.E., Mo H., Gao Y., Raina S.A., Zhang G.G.Z., Taylor L.S. (2017). Origin of Nanodroplet Formation Upon Dissolution of an Amorphous Solid Dispersion: A Mechanistic Isotope Scrambling Study. J. Pharm. Sci..

[14] Harmon P., Galipeau K., Xu W., Brown C., Wuelfing W.P. (2016). Mechanism of Dissolution-Induced Nanoparticle Formation from a Copovidone-Based Amorphous Solid Dispersion. Mol. Pharm..

[15] Stewart A.M., Grass M.E., Brodeur T.J., Goodwin A.K., Morgen M.M., Friesen D.T., Vodak D.T. (2017). Impact of Drug-Rich Colloids of Itraconazole and HPMCAS on Membrane Flux in Vitro and Oral Bioavailability in Rats. Mol. Pharm..

[16] Que C., Lou X., Zemlyanov D.Y., Mo H., Indulkar A.S., Gao Y., Zhang G.G.Z., Taylor L.S. (2019). Insights into the Dissolution Behavior of Ledipasvir-Copovidone Amorphous Solid Dispersions: Role of Drug Loading and Intermolecular Interactions. Mol. Pharm..

[17] Saboo S., Kestur U.S., Flaherty D.P., Taylor L.S. (2020). Congruent Release of Drug and Polymer from Amorphous Solid Dispersions: Insights into the Role of Drug-Polymer Hydrogen Bonding, Surface Crystallization, and Glass Transition. Mol. Pharm..

[18] Lakshman J.P., Cao Y., Kowalski J., Serajuddin A.T. (2008). Application of melt extrusion in the development of a physically and chemically stable high-energy amorphous solid dispersion of a poorly water-soluble drug. Mol. Pharm..

[19] Lang B., Liu S., McGinity J.W., Williams R.O. (2016). Effect of hydrophilic additives on the dissolution and pharmacokinetic properties of itraconazole-enteric polymer hot-melt extruded amorphous solid dispersions. Drug Dev. Ind. Pharm..

[20] Lu Y., Chen J., Yi S., Xiong S. (2019). Enhanced felodipine dissolution from high drug loading amorphous solid dispersions with PVP/VA and sodium dodecyl sulfate. J. Drug Deliv. Sci. Technol..

[21] Chen J., Ormes J.D., Higgins J.D., Taylor L.S. (2015). Impact of surfactants on the crystallization of aqueous suspensions of celecoxib amorphous solid dispersion spray dried particles. Mol. Pharm..

[22] Mosquera-Giraldo L.I., Trasi N.S., Taylor L.S. (2014). Impact of surfactants on the crystal growth of amorphous celecoxib. Int. J. Pharm..

[23] Meng F., Ferreira R., Zhang F. (2019). Effect of surfactant level on properties of celecoxib amorphous solid dispersions. J. Drug Deliv. Sci. Technol..

[24] Frank K.J., Westedt U., Rosenblatt K.M., Holig P., Rosenberg J., Magerlein M., Fricker G., Brandl M. (2012). The amorphous solid dispersion of the poorly soluble ABT-102 forms nano/microparticulate structures in aqueous medium: Impact on solubility. Int. J. Nanomed..

[25] Baghel S., Cathcart H., O’Reilly N.J. (2018). Investigation into the Solid-State Properties and Dissolution Profile of Spray-Dried Ternary Amorphous Solid Dispersions: A Rational Step toward the Design and Development of a Multicomponent Amorphous System. Mol. Pharm..

[26] Janssens S., Nagels S., Armas H.N., D’Autry W., Van Schepdael A., Van den Mooter G. (2008). Formulation and characterization of ternary solid dispersions made up of Itraconazole and two excipients, TPGS 1000 and PVPVA 64, that were selected based on a supersaturation screening study. Eur. J. Pharm. Biopharm..

[27] Feng D., Peng T., Huang Z., Singh V., Shi Y., Wen T., Lu M., Quan G., Pan X., Wu C. (2018). Polymer-Surfactant System Based Amorphous Solid Dispersion: Precipitation Inhibition and Bioavailability Enhancement of Itraconazole. Pharmaceutics.

[28] Karavas E., Georgarakis M., Docoslis A., Bikiaris D. (2007). Combining SEM, TEM, and micro-Raman techniques to differentiate between the amorphous molecular level dispersions and nanodispersions of a poorly water-soluble drug within a polymer matrix. Int. J. Pharm..

[29] Konno H., Handa T., Alonzo D.E., Taylor L.S. (2008). Effect of polymer type on the dissolution profile of amorphous solid dispersions containing felodipine. Eur. J. Pharm. Biopharm..

[30] Sarpal K., Delaney S., Zhang G.G.Z., Munson E.J. (2019). Phase Behavior of Amorphous Solid Dispersions of Felodipine: Homogeneity and Drug–Polymer Interactions. Mol. Pharm..

[31] Saboo S., Taylor L.S. (2017). Water-induced phase separation of miconazole-poly (vinylpyrrolidone-co-vinyl acetate) amorphous solid dispersions: Insights with confocal fluorescence microscopy. Int. J. Pharm..

[32] Grimaudo M.A., Pescina S., Padula C., Santi P., Concheiro A., Alvarez-Lorenzo C., Nicoli S. (2018). Poloxamer 407/TPGS Mixed Micelles as Promising Carriers for Cyclosporine Ocular Delivery. Mol. Pharm..

[33] Bhardwaj V., Trasi N.S., Zemlyanov D.Y., Taylor L.S. (2018). Surface area normalized dissolution to study differences in itraconazole-copovidone solid dispersions prepared by spray-drying and hot melt extrusion. Int. J. Pharm..

[34] Yang R., Mann A.K.P., Van Duong T., Ormes J.D., Okoh G.A., Hermans A., Taylor L.S. (2021). Drug Release and Nanodroplet Formation from Amorphous Solid Dispersions: Insight into the Roles of Drug Physicochemical Properties and Polymer Selection. Mol. Pharm..

[35] Li N., Taylor L.S. (2019). Microstructure Formation for Improved Dissolution Performance of Lopinavir Amorphous Solid Dispersions. Mol. Pharm..

[36] Han Y.R., Ma Y., Lee P.I. (2019). Impact of phase separation morphology on release mechanism of amorphous solid dispersions. Eur. J. Pharm. Sci..

[37] Li N., Cape J.L., Mankani B.R., Zemlyanov D.Y., Shepard K.B., Morgen M.M., Taylor L.S. (2020). Water-Induced Phase Separation of Spray-Dried Amorphous Solid Dispersions. Mol. Pharm..

[38] Robard A., Patterson D., Delmas G. (1977). The “Δη Effect” and Polystyrene-Poly(vinyl methyl ether) Compatibility in Solution. Macromolecules.

[39] Pouchlý J., Patterson D. (1976). Polymers in Mixed Solvents. Macromolecules.

[40] Surana R., Randall L., Pyne A., Vemuri N.M., Suryanarayanan R. (2003). Determination of glass transition temperature and in situ study of the plasticizing effect of water by inverse gas chromatography. Pharm. Res..

[41] Chen H., Pui Y., Liu C., Chen Z., Su C.C., Hageman M., Hussain M., Haskell R., Stefanski K., Foster K. (2018). Moisture-Induced Amorphous Phase Separation of Amorphous Solid Dispersions: Molecular Mechanism, Microstructure, and Its Impact on Dissolution Performance. J. Pharm. Sci..

[42] Purohit H.S., Taylor L.S. (2017). Phase Behavior of Ritonavir Amorphous Solid Dispersions during Hydration and Dissolution. Pharm. Res..

[43] Ueda K., Taylor L.S. (2020). Polymer Type Impacts Amorphous Solubility and Drug-Rich Phase Colloidal Stability: A Mechanistic Study Using Nuclear Magnetic Resonance Spectroscopy. Mol. Pharm..

[44] Mosquera-Giraldo L.I., Li N., Wilson V.R., Nichols B.L.B., Edgar K.J., Taylor L.S. (2018). Influence of Polymer and Drug Loading on the Release Profile and Membrane Transport of Telaprevir. Mol. Pharm..

[45] Rangel-Yagui C.O., Pessoa J.A., Tavares L.C. (2005). Micellar solubilization of drugs. J. Pharm. Pharm. Sci..

[46] Vinarov Z., Katev V., Radeva D., Tcholakova S., Denkov N.D. (2018). Micellar solubilization of poorly water-soluble drugs: Effect of surfactant and solubilizate molecular structure. Drug Dev. Ind. Pharm..

[47] Miller-Chou B.A., Koenig J.L. (2003). A review of polymer dissolution. Prog. Polym. Sci..

[48] Craig D.Q. (2002). The mechanisms of drug release from solid dispersions in water-soluble polymers. Int. J. Pharm..

[49] Wu J. (1992). Solvent Diffusion and Dissolution of Glassy Polymers. Ph.D. Thesis.

