# Supplementary material for: Exploring the Role of Surfactants in Enhancing Drug Release from Amorphous Solid Dispersions at Higher Drug Loadings
Source: Pharmaceutics. 2021 May 17;13(5):735. doi: 10.3390/pharmaceutics13050735 (PMC8156319; doi:10.3390/pharmaceutics13050735)
Supplement: Supplementary file 1 [file pharmaceutics-13-00735-s001.zip › pharmaceutics-1202288-supplementary.pdf]

Supplementary Materials: Exploring the Role of Surfactants in Enhancing Drug Release from Amorphous Solid Dispersions at Higher Drug Loadings

Sugandha Saboo [1,2,†], Pradnya Bapat [1,†], Dana E. Moseson [1], Umesh S. Kestur [3] and Lynne S. Taylor [1,*]

Table S1. Physicochemical and thermodynamic properties of felodipine.*

Parameter	Value
Molecular weight (g/mol)	384.25
T_m (°C)	147
T_g (°C)	45
log P	3.44
Enthalpy of fusion (kJ/mol)	28.67
H-bond donors	1
H-bond acceptors	3
Rotatable bonds	6
Polar surface area (Å^2)	65

*Values are referenced from: 1. Raina, S. A.; Van Eerdenbrugh, B.; Alonzo, D. E.; Mo, H.; Zhang, G. G. Z.; Gao, Y.; Taylor, L. S. Trends in the precipitation and crystallization behavior of supersaturated aqueous solutions of poorly water-soluble drugs assessed using synchrotron radiation. *J Pharm Sci* **2015,** *104*, (6), 1981-1992.

2. Raina, S. A.; Zhang, G. G. Z.; Alonzo, D. E.; Wu, J.; Zhu, D.; Catron, N. D.; Gao, Y.; Taylor, L. S. Enhancements and limits in drug membrane transport using supersaturated solutions of poorly water soluble drugs. J Pharm Sci 2014, 103, (9), 2736-2748.

Table S2. ASD compositions studied/referenced in this work. All percent (%) compositions refer to weight%. TPGS% is on a weight percent basis out of the polymer composition. Values in red refer to polymer weight % and values in green refer to TPGS concentration (g/mL) in the dissolution medium (100 mL) assuming 100% release.

50%				45% / 50
45%				49.5% / 55
40%			57% / 30	54% / 60
35%			61.75% / 32.5	
30%	70% / 0	68.6% / 14	66.5% / 35	63% / 70
25%		73.5% / 15		
20%	80% / 0	78.4% / 16	76% / 40	72% / 80
15%	85% / 0			
10%	90% / 0	88.2% / 18	85.5% / 45	81% / 90
5%	95% / 0			
DL (%) / TPGS (%)	0%*	2%	5%	10%

*Values corresponding to 0% TPGS ASDs are referenced from, "Saboo, S.; Moseson, D. E.; Kestur, U. S.; Taylor, L. S. Patterns of drug release as a function of drug loading from amorphous solid dispersions: A comparison of five different polymers. European Journal of Pharmaceutical Sciences 2020, 155, 105514".

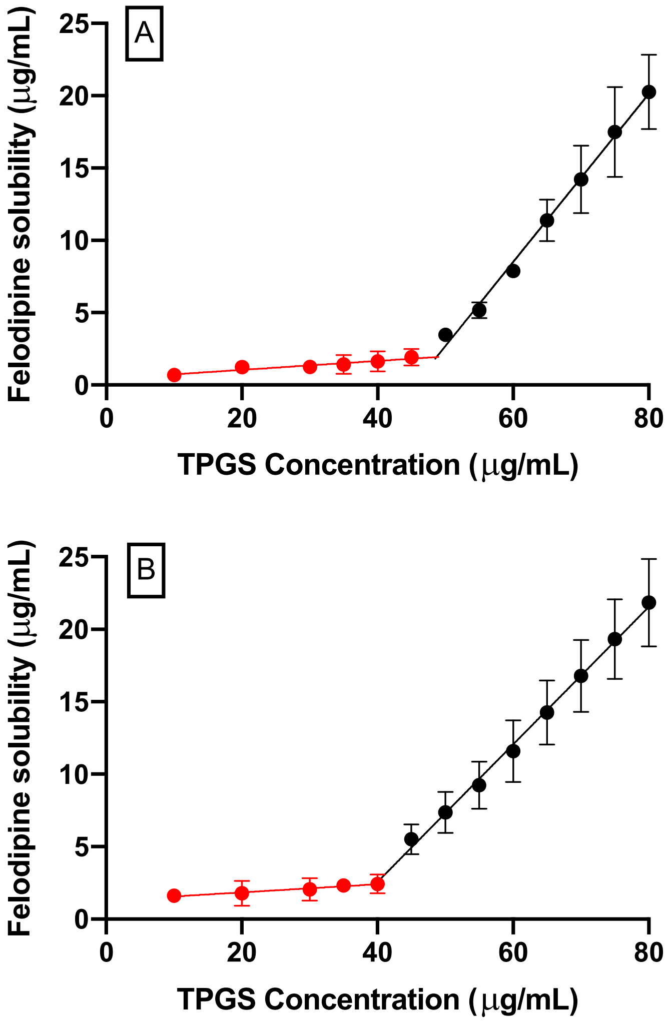

Figure S1. CMC of TPGS as determined by a change in slope of crystalline felodipine solubility in pH 6.8 buffer as a function of different concentrations of TPGS. **(A)** In the absence of PVPVA. (**B**) In the presence of 900 μg/mL pre-dissolved PVPVA. Error bars represent standard deviations (n = 3).

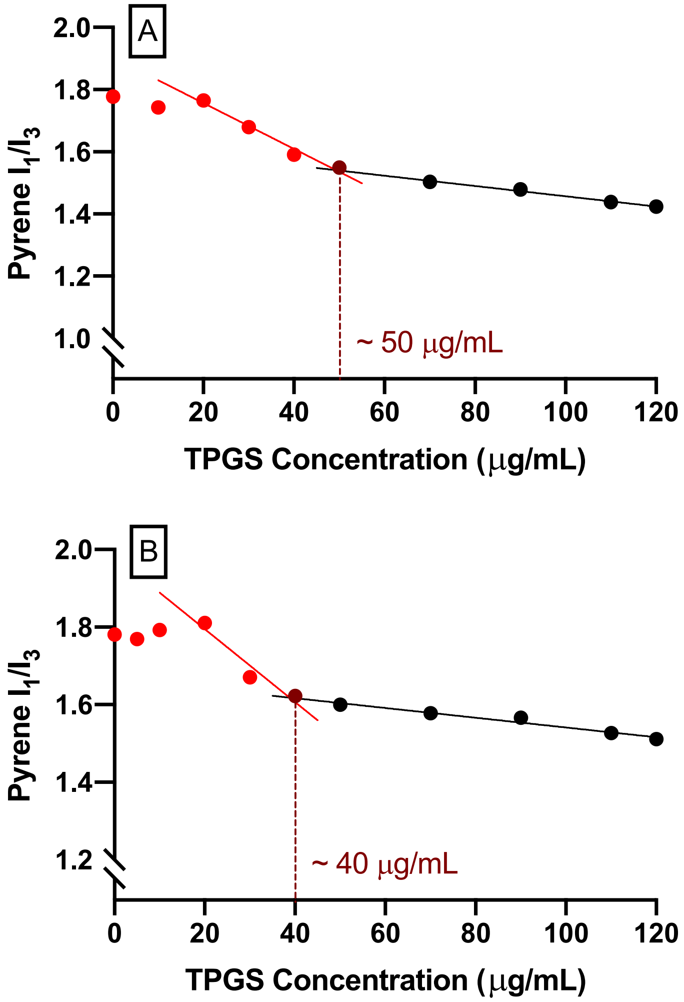

Figure S2. TPGS CMC determination in pH 6.8 phosphate buffer by the pyrene fluorescence method. CMC is determined by the change of slope. (**A**) In the absence of PVPVA. (**B**) In the presence of 900 μg/mL pre-dissolved PVPVA.

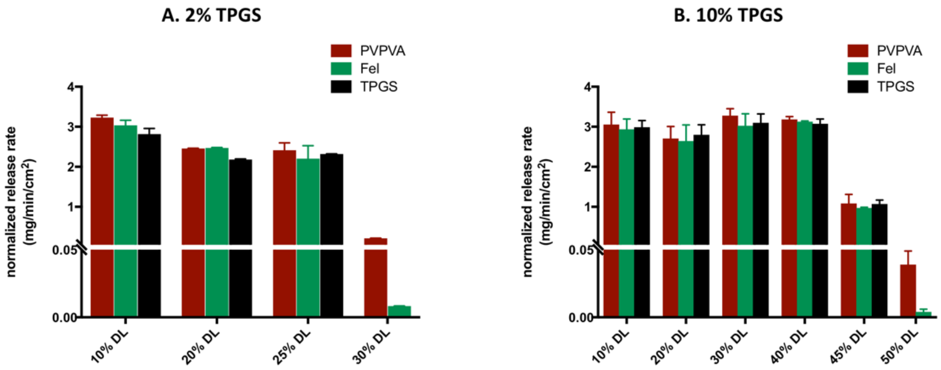

Figure S3. Surface normalized dissolution rates for amorphous felodipine, PVPVA, and TPGS for different drug loading ASDs with 2% TPGS (**A**) and 10% TPGS (**B**). Error bars represent standard deviations, $n = 3$.

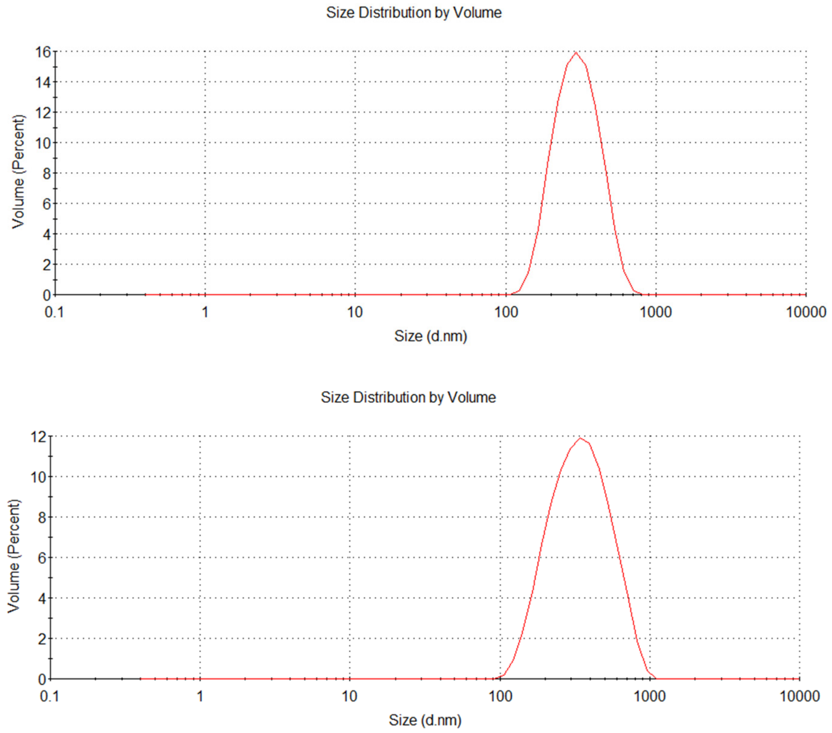

Figure S4. Representative DLS data showing size distribution for amorphous nanoparticles generated upon dissolution: without TPGS (top) and with TPGS (5%) (bottom) at 10% DL.

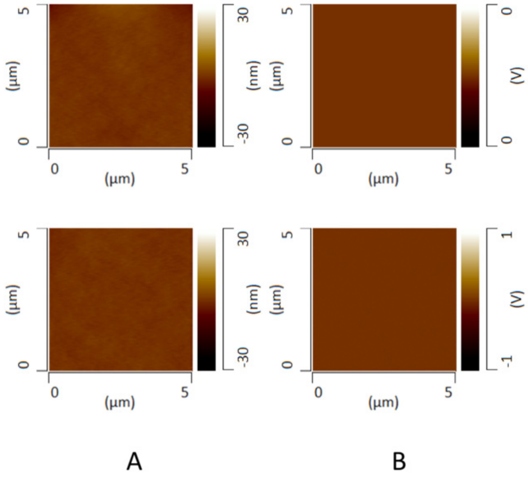

Figure S5. Representative AFM topographical (**A**) and deflection images (**B**) of the freshly prepared films of binary felodipine-PVPVA ASD (top) and ternary felodipine-PVPVA ASD at 10% TPGS level (bottom).
